# Split-Framework in Mandibular Implant-Supported Prosthesis

**DOI:** 10.1155/2015/502394

**Published:** 2015-12-03

**Authors:** Danny Omar Mendoza Marin, Kássia de Carvalho Dias, André Gustavo Paleari, Ana Carolina Pero, João Neudenir Arioli Filho, Marco Antonio Compagnoni

**Affiliations:** ^1^Department of Dental Materials and Prosthodontics, Araraquara Dental School, Universidade Estadual Paulista (UNESP), Humaitá Street 1680, 14801-903 Araraquara, SP, Brazil; ^2^Department of Restorative Dentistry, Alfenas Dental School, Federal University of Alfenas, Alfenas, MG, Brazil

## Abstract

During oral rehabilitation of an edentulous patient with an implant-supported prosthesis, mandibular flexure must be considered an important biomechanical factor when planning the metal framework design, especially if implants are installed posterior to the interforaminal region. When an edentulous mandible is restored with a fixed implant-supported prosthesis connected by a fixed full-arch framework, mandibular flexure may cause needless stress in the overall restorative system and lead to screw loosening, poor fit of prosthesis, loss of the posterior implant, and patient's discomfort due to deformation properties of the mandible during functional movements. The use of a split-framework could decrease the stress with a precise and passive fit on the implants and restore a more natural functional condition of the mandible, helping in the longevity of the prosthesis. Therefore, the present clinical report describes the oral rehabilitation of an edentulous patient by a mandibular fixed implant-supported prosthesis with a split-framework to compensate for mandibular flexure.* Clinical Significance.* The present clinical report shows that the use of a split-framework reduced the risk of loss of the posterior implants or screws loosening with acceptable patient comfort over the period of a year. The split-framework might have compensated for the mandibular flexure during functional activities.

## 1. Introduction

The oral rehabilitation of an edentulous patient treated with a fixed implant-supported prosthesis using appropriate biomechanical and prosthetic principles has been a goal in oral implant research for the last decade [1]. With the fixed implant-supported prosthesis, an adequate distribution of stress is very important to decrease implant and prosthetic failures [2]. In addition, these failures can also be influenced by several factors, including prosthetic design and occlusal scheme [3].

A common treatment for an edentulous mandible is the installation of implants in the interforaminal region and a full-arch fixed implant-supported prosthesis with cantilever distal extensions [1, 4]. However, this type of prosthesis can promote a high level of stress that can be harmful to the implant and the surrounding bone as a result of the unfavorable lever arms [5, 6]. For this reason, it has been suggested that the use of multiple implants in the anterior and posterior mandible could improve the distribution of stress with more favorable implant support, avoiding long cantilevers [7].

Although it is an alternative, the use of an implant fixed restoration with a continuous and rigid framework can create dangerous stress at the bone/implant interface and at the prosthetic superstructure due to the deformation properties of the mandible, which can occur during functional and parafunctional loads [8, 9]. Mandibular flexure is defined as “the change in shape of the mandible caused by the pterygoid muscles contracting during opening and protrusion movements” [10] and can affect the biomechanical behavior, passive fit, and long-term prognosis of the fixed implant-supported prosthesis if it is not considered [11].

During functional activities, mandibular flexure should be considered an important biomechanical factor in the design of a partial or complete fixed implant-supported prosthesis in the mandible with dental implants [12–16]. In natural dentition, the stress generated by mandibular flexure can be compensated by adaptation of the periodontal ligament. However, this stress in a fixed implant-supported prosthesis is transmitted around the mandibular bone and could induce stress increases in the implant-related prosthesis and abutments and cause damage to the bone-implant interface [1, 12, 15], especially in posterior implants [17], bone loss around the implant, loss of implant fixation, material fracture, and/or loss of retention of restorations [12].

Therefore, for better longevity and outcomes of implant-related prostheses, it is important to reduce the effects of mandibular flexure [14, 16]. Several designs of mandibular fixed implant-supported prostheses have been suggested to improve the distribution of stress resulting from mandibular flexure [8, 12, 18]. The aim of the present case report is to describe the oral rehabilitation of an edentulous patient by a mandibular fixed implant-supported prosthesis with a split-framework to compensate for mandibular flexure.

## 2. Case Description

**Figure 1:**
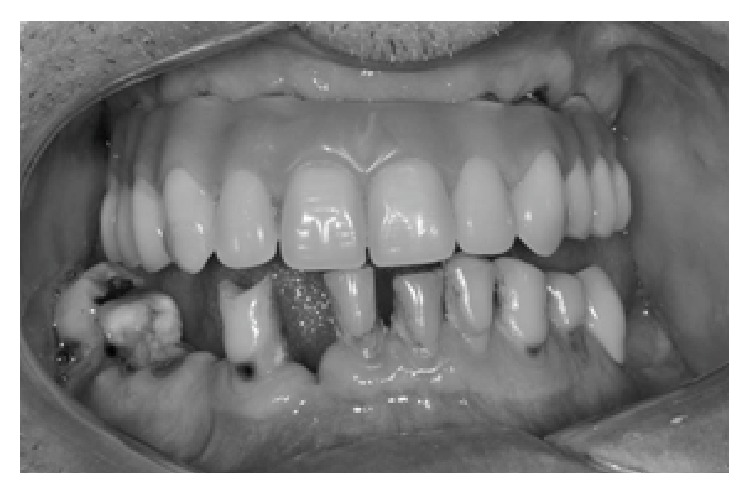
Initial aspect of the patient before treatment.

A 51-year-old man was referred to the Araraquara Dental School, UNESP, Universidade Estadual Paulista, Araraquara, SP, Brazil, for assessment and manufacture of a mandibular fixed implant-supported prosthesis. The main complaint was mobility and pain of the remaining teeth in the mandible. An intraoral examination was performed and the presence of caries in some teeth was verified, as well as chronic periodontitis in all remaining teeth (Figure 1). The patient was also a wearer of a maxillary fixed implant-supported prosthesis.

**Figure 2:**
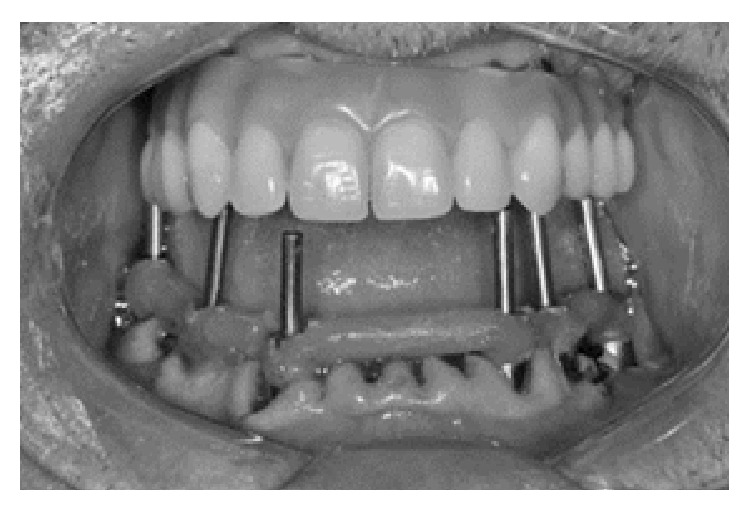
Impression of the abutments with the impression coping after extraction of the remaining teeth of the mandible.

The proposed treatment for the mandible was extraction of the remaining teeth, installation of six implants, and immediate loading with a mandibular fixed implant-supported prosthesis. After the extraction of the remaining teeth, four implants were installed in the posterior mandible and two implants were installed in the interforaminal region (Titanium Ti Cortical, Ø 3.75 mm, Neodent, Curitiba, Brazil). Six minicone abutments (Mini Cone Abutment, Neodent, Curitiba, Brazil) were also installed and the impression was performed using impression coping (Impression Coping Mini Cone Abutment, Neodent, Curitiba, Brazil) splinted with polymethyl methacrylate (PMMA) resin (Duralay, Reliance Dental, IL, USA) (Figure 2) and polydimethylsiloxane (Zhermack, Zetaplus, Badia Polesine, Italy). The impression was poured with special plaster type IV (Vel-Mix Stone, Kerr Corporation, Orange, California, USA) and a record base with an occlusion rim was used to establish the occlusal vertical dimension and record patient's centric relation [19].

Finally, the definitive casts were mounted in a semiadjustable articulator (Bio-Art Equipamentos Odontológicos Ltda.©, São Carlos, São Paulo, Brazil) and artificial acrylic resin teeth (Biotone, Dentsply Ind. e Com. Ltda., Rio de Janeiro, RJ, Brazil) were set and evaluated in the patient. A framework was manufactured using a castable coping of abutments and Ni-Cr alloy (Fit Cast SB-Plus Ni-Cr without Beryllium, Talladium, Curitiba, PR, Brazil).

Next, a simplified technique for fixed implant-supported prosthesis was used for the wax teeth and framework trials [20]. In this technique, the wax teeth were prepared on a light-polymerized resin base, and both the wax teeth and framework trials were accomplished in the same session. Following this, a framework try-in on the abutments was performed by first tightening down one of the terminal screws completely on the right side. After clinical and radiographic verification, the screw was unscrewed and the procedure was repeated for the other terminal abutment [21, 22] to verify the passive fit. Later, the teeth in the wax on the base of light-polymerized resin were tested on the framework during the same clinical session.

**Figure 3:**
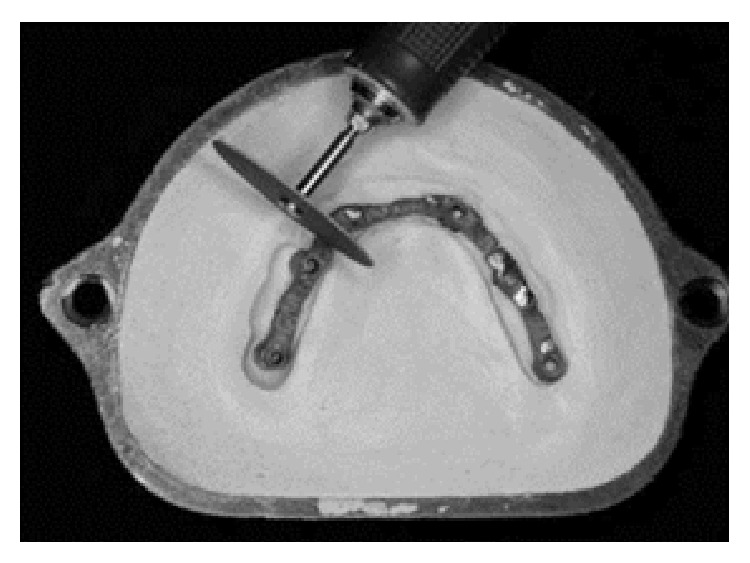
Splitting of the framework with a carborundum disc.

**Figure 4:**
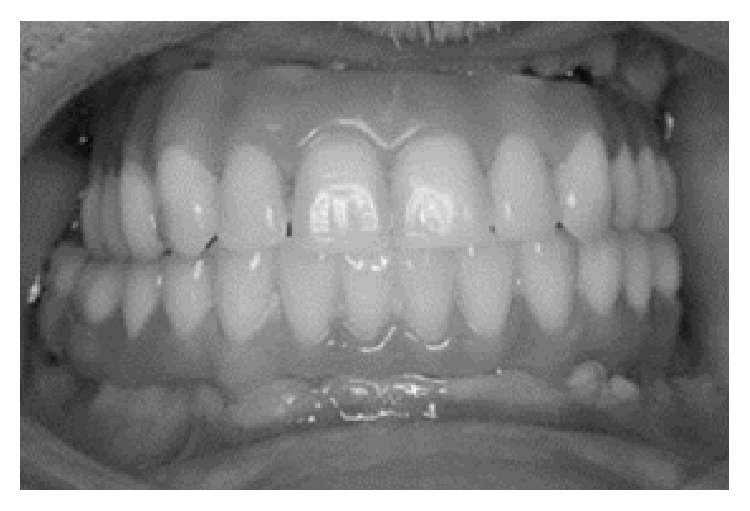
Installation of mandibular implant-supported prosthesis.

**Figure 5:**
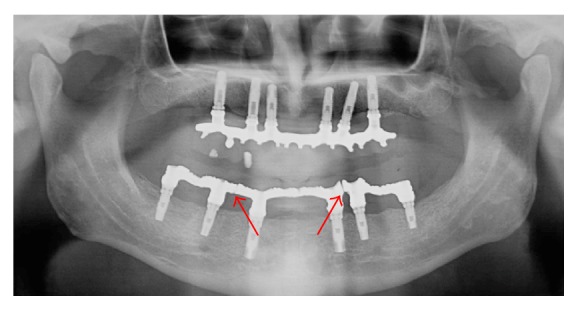
Panoramic radiograph after a year of installation of the mandibular fixed implant-supported prosthesis. Red arrows show the cuts in framework.

After intraoral assessment, the framework within the teeth was included in a flask (Flask, OGP Produtos Odontológicos Ltda., São Paulo, SP, Brazil) with plaster type III (stone plaster type III, Vigodent, Rio de Janeiro, RJ, Brazil). The wax was removed and the framework was split into three sections with a carborundum disc (Figure 3). Heat-polymerized polymethyl methacrylate resin (Lucitone 550, Dentsply International Inc., New York, USA) was used for manufacturing of the mandibular fixed implant-supported prosthesis. The mandibular fixed implant-supported prosthesis was installed in the mouth (Figure 4), occlusal adjustment was performed, and a panoramic radiograph was taken. One year after placement, the patient did not present any complaint, loss of posterior implants, or screw loosening.  Figure 5 shows a panoramic radiograph after a year.

## 3. Discussion

Achieving lower stress in an implant-supported restorative system is one of the main goals of implant treatment from a mechanical point of view. In the present case, the framework was split in three pieces (Figures 3 and 5) because lower stress is present during molar clenching [12], because precise and passive fit could be achieved decreasing the stress around the implants [23], and because it is difficult to fit a fixed full-arch framework passively against the abutments by casting alone [24]. Moreover, the division of the superstructure into shorter segments seemed to restore a more natural functional condition of the mandible [1].

During oral rehabilitation of edentulous patients with mandibular fixed implant-supported prostheses, mandibular flexure could be affected by two important factors: (1) position of the implants and (2) typology of the prosthetic structure [1]. When additional implants are installed beyond the interforaminal region, they are associated with a higher risk of loss, probably due to the deformation of the mandible during functional movement [17]. Also, the use of a fixed full-arch framework to connect all implants could reduce the mandibular flexure and consequently increase the stress in the bone around the implants [1] and induce their premature failure [25].

Furthermore, the mandibular flexure must be considered an important factor because it could contribute to discomfort related to the patients' rehabilitation with a mandibular fixed implant-supported prosthesis during function [26]. In this case, the recovery from the pain and symptoms could be achieved only after splitting the prosthesis into three sections [26], possibly due to decreased stress on mandibular flexion with this prosthesis design. Therefore, for better longevity and outcomes of implant-related prosthesis, it is important to reduce the effect of mandibular flexure [15, 16, 23].

A study of stress analysis has shown that frameworks constructed with a precise and passive fit induce significantly smaller amounts of stress on the implant [23] and this could be achieved by sectioning the framework into small pieces, improving the passive fit [11]. Thus, the section of the framework could decrease the stress on the implant during functional movement of the mandible, especially when posterior implants are installed behind the mental foramen, increasing their longevity.

Sectioning the prostheses into two or three pieces [12, 18] has been recommended to allow mandibular flexure of the restored mandible to come close to its natural state [1, 11]. It has been hypothesized that these designs will minimize stress concentration in posterior and anterior implants [12]. However, despite the biomechanical advantages of these fixed implant-supported prostheses designs, the aesthetic is affected by the sectioning of the final prosthesis and these sections could lead food to impact on the sectioned areas, compromising the patient's hygiene.

The contraction of the lateral pterygoid muscle is the most important factor causing mandibular deformation during function and four patterns of jaw deformation were postulated: symphyseal bending, dorsoventral shear, corporal rotation, and anteroposterior shear [27]. Splitting the framework into two pieces at the mandibular midline could decrease the stress during symphyseal bending but it does not prevent the forces generated by corporal rotation and could produce strains in the complex implant-prosthesis. Therefore, splitting the framework into three pieces has better advantages to decrease the effect of mandibular rotation, providing better treatment outcomes.

Considering that the modulus of elasticity of the PMMA resin is lower than the framework of Ni-Cr and due to the sectioning of the framework, the PMMA resin could suffer deformation during mandibular flexure, decreasing stress. Furthermore, because the framework was split into three pieces, it could be suggested that the stress during mandibular flexure is lower when compared with an implant-supported prosthesis with a fixed full-arch framework.

In the present case, the installation of posterior implants was used to decrease the lever arm, allowing greater posterior extension and increased occlusion scheme in the mandibular fixed implant-supported prosthesis, which provided a better distribution of occlusal forces and increased the prosthesis stability. In addition, the precise and passive fit of the split-framework and properties of PMMA resin could have reduced the effect of the mandibular flexure damage in the bone-implant interface in the posterior implants, which may help to increase the longevity of the prosthesis.

## 4. Conclusions

The use of a mandibular fixed implanted-supported prosthesis with a split-framework is a good alternative to compensate for mandibular flexure, providing good stability and retention of the implant-supported prosthesis without loss of the posterior implant or screw loosening with acceptable patient comfort during a period of one year.
